# *Lactobacillus fermentum* MCC2759 and MCC2760 Alleviate Inflammation and Intestinal Function in High-Fat Diet-Fed and Streptozotocin-Induced Diabetic Rats

**DOI:** 10.1007/s12602-021-09744-0

**Published:** 2021-02-11

**Authors:** Ann Catherine Archer, Serva Peddha Muthukumar, Prakash Motiram Halami

**Affiliations:** 1 Microbiology and Fermentation Technology Department, Mysuru, India; 2 Department of Biochemistry, CSIR-Central Food Technological Research Institute, 570020 Mysuru, India

**Keywords:** Probiotics, *Lactobacillus* spp., IL-10, GLP-1, Streptozotocin (STZ)-induced diabetes

## Abstract

The growing incidence of type 2 diabetes and obesity has become a worldwide crisis with increased socio-economic burden. Changes in lifestyle and food habits resulting in dysbiosis of the gut microbiota and low-grade inflammation are linked to the rising incidence. The aim of this study was to investigate the effects of potential probiotic *Lactobacillus fermentum* MCC2759 and MCC2760 on intestinal markers of inflammation using a high-fat diet (HFD)-fed model and a streptozotocin (STZ)-induced diabetic model. *Lact. fermentum* administration showed improved oral glucose tolerance compared with the model controls of HFD (AUC 1518) and STZ (628.8). Plasma insulin levels improved in the *Lact. fermentum* treated groups of HFD + MCC2759 (129 ± 4.24 pmol/L) and HFD + MCC2760 (151.5 ± 9.19 pmol/L) in HFD study, while in STZ diabetic study, the insulin levels were normalized with *Lact. fermentum* administration, for D + MCC2759 (120.5 ± 7.77) and D + MCC2760 (138 ± 5.65 pmol/L) groups. The results showed reduction in inflammatory tone in liver, muscle, and adipose tissues of rats in both models with stimulation of anti-inflammatory IL-10 by real-time quantitative polymerase chain reaction. Additionally, the potential probiotic cultures also displayed normalization of markers related to intestinal barrier integrity (ZO-1), TLR-4 receptor, and insulin sensitivity (GLUT-4, GLP-1, adiponectin). Thus, the results suggest that *Lact. fermentum* could act as potential probiotic for lifestyle-related disorders such as obesity, diabetes, and metabolic syndrome as both prophylactic and adjunct therapies.

## Introduction

Over the previous decades, there is an unabated increase in the incidence of diabetes and obesity worldwide. Diabetes is a multifactorial disease accounting for about 3.5% of mortality due to non-communicable diseases [1]. The incidence of diabetes is said to increase to 592 million worldwide by 2035 with an estimated high burden of disease prevalence in developing countries. Moreover, most diabetic individuals are obese [2]. Consumption of high-fat foods is a crucial factor for the rise in metabolic diseases and diabetes. Several environmental and genetic factors together with low lying inflammation contribute to the development of insulin resistance coupled with dysbiosis of the gut microbiota leading to the onset of diabetes. This process occurs by host receptors that recognize molecular microbial patterns such as TLRs (toll-like receptors) and NLRs (nucleotide oligomerization domain (NOD)-like receptors) through which bacteria and their components stimulate the immune response [3]. The inflammation is characterized by increased secretion of TNF-α and other pro-inflammatory cytokines which is known to activate protein kinase C (PKC) leading to enhanced phosphorylation of insulin receptor substrates and its subsequent inactivation, ultimately culminating in insulin resistance [4]. However, the antigens responsible for the induction of inflammation were not known earlier.

The human gastrointestinal tract (GIT) is composed of more than 500 bacterial species, 90% of which belong to Bacteroidetes (Gram-negative bacteria) and Firmicutes (Gram-positive bacteria). The role of intestinal microbiota has recently been implicated in conditions such as gastrointestinal inflammatory disorders, allergies, cardiovascular diseases, cancer, and diabetes [5]. Dysbiosis of the Gram-negative and Gram-positive bacteria ratio increases barrier permeability and translocation of bacterial lipopolysaccharide (LPS). This bacterial translocation is thought to cause metabolic endotoxemia, trigger the mucosal immune response, cause insulin resistance, and eventually diabetes [6]. Diabetic and obese individuals show disturbances in the compositions of Firmicutes, Bacteroidetes, and Proteobacteria. Diabetics are found to have lower counts of *Bifidobacterium* and *Faecalibacterium prausnitzii*, both of which are known to possess anti-inflammatory properties [7]. Studies of high-fat diet-fed (HFD) animals have shown dysbiosis of the intestinal microbiota coupled by translocation of bacteria from the intestine to other tissues triggering localized inflammation. High-fat feeding also showed an increase in the Gram-negative bacteria to Gram-positive bacteria ratio [3, 7]. Vrieze et al. [8] demonstrated improvement in insulin sensitivity of patients suffering from metabolic syndrome post microbiota administration from lean subjects.

Although lifestyle changes are the primary therapeutic approach for obesity and metabolic syndrome, research for novel therapies targeting the underlying conditions is being probed. Recently, probiotics are known to reduce high-fat diet-induced obesity, diabetes, and metabolic syndrome [9]. Supplementation of probiotics is known to restore the gut microbial balance and modulate the immune response. Probiotics have been suggested as effective adjuvants for the treatment of insulin resistance and are explored as therapeutic strategies for the prevention of diabetes onset. Strains of *Lactobacillus* and *Bifidobacterium* have shown beneficial effects for glucose equilibrium, hepatic steatosis, and inflammation reduction [10, 11]. Lactobacilli are also known to possess strong immunomodulatory and anti-inflammatory properties. Hence, the ability of native *Lactobacillus* cultures to prevent the onset of high-fat diet-induced diabetes through immunomodulation needs to be explored.

HFD model depicts characteristics of low-grade inflammation onset, while a high-fat diet along with low-dose streptozotocin (STZ) mimics metabolic characteristics of type 2 diabetes [3, 12]. Probiotic effects are strain-specific. Hence, to obtain a potential probiotic with anti-inflammatory activity, we screened several fermented dairy products and infant faecal samples and selected two strains of *Lact. fermentum*, MCC2759 (fecal isolate) and MCC2760 (curd isolate). The probiotic properties in vitro in addition to strong adhesion and anti-inflammatory activity in cell line and carrageenan-induced rat model were previously studied [13–15]. In this study, we aimed to study the effects of these two isolates in two models. HFD model will address whether potential probiotics can prevent lifestyle-induced pre-diabetic or diabetic condition via an anti-inflammatory effect. STZ-induced type 2 diabetes model will investigate the therapeutic or adjunct benefits in diabetic conditions.

## Materials and Methods

### Bacterial Cultures

Potential probiotic isolates *Lact. fermentum* MCC2759 and *Lact. fermentum* MCC2760 identified in our laboratory were used in this study. Viable count of overnight grown cultures (1.5 OD) was taken by serial dilution and plating on MRS agar. *Lact. fermentum* MCC2759 and *Lact. fermentum* MCC2760 had approximately 10^9^ CFU/ml. The cultures were propagated in MRS medium overnight, and 10^9^ CFU/ml of cells were suspended in phosphate-buffered saline (PBS) for oral administration in rats.

### Animal Maintenance and Diet

Wistar rats (female) weighing 120–160 g were housed at the institute Animal House Facility, CSIR-Central Food Technological Research Institute, Mysuru. The animals were maintained under controlled temperature and humidity with a 12-h light and dark cycle in standard polypropylene cages. The rats were acclimatized for a week, fed with a normal diet (AIN 93G), and had access to food and water ad libitum. The experimental procedures were followed as per the guidelines of Committee for the Purpose of Control and Supervision of Experiments on Animals (CPCSEA), Government of India, and approved by the Institute Animal Ethical Committee (IAEC), CSIR-CFTRI, Mysuru, following IAEC regulations (Approval no. 335/14).

#### Study 1: High-Fat Diet Feeding and Grouping

All animals were fed a normal diet (AIN 93G) for a week before dietary manipulation. Thereafter, normal control rats were fed with a normal diet, while experimental groups received a high-fat diet (Supplementary data 1) for an initial period of 4 weeks. The animals had access to water ad libitum. Subsequently, the rats were divided into the following groups (*n* = 6 rats per group): (1) normal control (NC), (2) high-fat diet control (HFDC), and (3) and (4) high-fat diet-fed groups treated intragastrically with potential probiotic *L. fermentum* MCC2759 and MCC2760 (10^9^ CFU/ml suspended in PBS) once daily for 4 weeks, respectively. The total duration of the study was 8 weeks.

#### Study 2: Induction of Type 2 Diabetes and Grouping

A total of 40 rats (120–160 g) were taken, control rats were fed a normal diet, while the remaining rats were fed an HFD for 4 weeks as described above. Overnight fasting HFD rats were injected with a single low dose of STZ (Sigma-Aldrich Pvt. Ltd., Bengaluru, India) at 45 mg/kg body weight dissolved in 0.1 M citrate buffer (pH 4.5) intraperitoneally. Normal or non-diabetic rats (*n* = 10) were administered with only citrate buffer as a vehicle. STZ-induced diabetic rats were given 5% glucose solution for the first 24 h to surmount drug-induced hypoglycaemia. Diabetic rats with fasting blood glucose of > 200 mg/dL were selected 7 days after streptozotocin injection. The rats (*n* = 10/group) were randomly divided into the following groups: (1) normal control (NC), (2) diabetic control (DC), and (3) and (4) diabetic rats treated with potential probiotic *Lact. fermentum* MCC2759 and *Lact. fermentum* MCC2760 (10^9^ CFU/ml) intragastrically for 4 weeks, respectively.

### Food Intake, Weight Gain, and Organ Weight

Food intake and gain in body weight were monitored weekly during the experimental period (4 weeks for type 2 diabetic study and 8 weeks for HFD fed study). The weight of different organs was taken post-necropsy.

### Oral Glucose Tolerance Test

Oral glucose tolerance test (OGTT) was performed after 8 weeks and 4 weeks of study 1 and study 2, respectively. Rats were starved for 12–14 h before intragastric gavage of glucose (200 g/L in solution; 2 g/kg body weight). Blood was taken by pricking the tail vein and measured using a glucometer (Accu-Chek, Roche Diabetes Care India Pvt. Ltd., Mumbai) at 0, 30, 60, and 120 min post glucose administration. Area under curve was calculated from the blood glucose values (mg/dL) to determine the glucose excursion or tolerance.

### Tissue Processing

Animals were euthanized after 4 weeks of experiment duration. Blood was collected by cardiac puncture and allowed to clot at 4 °C for 2 h followed by centrifugation at 757 g-force for 20 min at 4 °C, and serum was collected. The serum was stored at − 20 °C until further analysis. Tissues of the intestine, liver, MAT (mesenteric adipose tissue) and muscle were collected and stored in RNA later solution (Sigma-Aldrich Pvt. Ltd., Bengaluru, India) until further use.

### Serum Biochemical Analysis

Levels of glucose, cholesterol, high-density lipoprotein (HDL-C), low-density lipoprotein (LDL-C), triglycerides, cholesterol, total protein, uric acid, urea, creatinine, albumin, SGPT, and SGOT were analyzed in the serum using standard analytical kits (Agappe India Pvt. Ltd., Kerala, India). Plasma insulin was determined using ELISA as per the manufacturer’s instructions (Mercodia, Uppsala, Sweden).

### Evaluation of Marker Genes by Real-Time qPCR

Intestine, liver, MAT, and muscle tissues (1 g) were homogenized in a tissue homogenizer. The homogenate was subjected to RNA isolation using 1-ml Trizol reagent (Sigma-Aldrich, Bengaluru, India) according to the manufacturer’s instructions. RNA was reversed transcribed into cDNA using Transcriptor High Fidelity cDNA Synthesis Kit (Bionova-Roche Chemicals, Bengaluru, India) as per manufacturer’s instructions. Real-time quantitative polymerase chain reaction (qPCR) (Biorad CFX-96, Bengaluru, India) was carried out using diluted cDNA (1:25) as a template, SYBR Green Jumpstart *Taq* ReadyMix (Sigma-Aldrich, Bengaluru, India), and gene-specific primers. The PCR program consisted of an initial denaturation at 94 °C for 3 min followed by 40 cycles of denaturation (94 °C for 30 s), annealing (60 °C for 30 s), and elongation (72 °C for 30 s). Primer specificity and efficiency were evaluated from the melt curves. Data were obtained from triplicate samples run along with no-template control. Oligonucleotide primers were designed using the Primer3 software (http://primer3.ut.ee/) by obtaining consensus sequences belonging to rat (*Rattus norvegicus*) genes from NCBI and later synthesized from Eurofins Genomics India Pvt Ltd (Bengaluru, India). The primers related to inflammatory cytokine genes and other markers are listed in Supplementary data 2. Glyceraldehyde 3-phosphate dehydrogenase (GAPDH) gene was used as an endogenous control for normalization of gene expression. Gene expression results were expressed as relative normalized expression (fold change) calculated using the 2^−^ΔΔCt method.

### Histopathology

Tissues of liver, kidney, muscle, pancreas, and intestine were sectioned and stained with hematoxylin and eosin (H&E) stain for histopathology evaluation. The stained tissue slides were observed under a bright-field microscope (Labomed, Burlington, NC, USA).

### Statistical Analysis

Data were statistically analyzed using GraphPad Prism version 5.00 for Windows (GraphPad Software, San Diego California USA, www.graphpad.com). All data were expressed as mean ± SD/SEM (*n* = 6). One-way ANOVA was used for the analysis of a single parameter, and two-way ANOVA analysis was used for comparison between groups. *p* < 0.05 and *p* < 0.001 were considered significant for one-way and two-way ANOVA, respectively.

## Results

### Food Intake, Weight Gain, and Organ Weight

Food intake of groups fed with a high-fat diet (study 1) increased significantly compared with the NC group. However, a slight decrease was seen in the groups administered with potential probiotics post 4 weeks of the experiment regimen. Similarly, an increase in body weight was observed in the groups fed a high-fat diet during the initial 4 weeks. However, groups treated with *Lact. fermentum* gradually showed reduced weight (203 g at 8th week) as opposed to the high-fat diet control group (225 g at 8th week) which continued to significantly gain weight throughout the experimental duration. Food intake and body weight changes are presented in Supplementary data 3. The weight of organs post-necropsy such as heart, lungs, liver, kidney, intestine, caecum, and stomach showed a substantial increase in the high-fat diet-fed control groups compared with the normal control rats. However, the weight of the organs seemed to be normalized in the groups supplemented with *Lact. fermentum* MCC2759 and MCC2760 (Table 1).

**Table 1:** Weight (g) of different organs of diabetic study groups and high-fat diet study groups post necropsy

Groups	Heart		Lungs	Liver	Pancreas	Spleen	Kidneys	Intestine	Cecum	Stomach	Ovaries	Adrenal glands
High-fat diet study 1												
NC		0.77 ± 0.04^b^	1.20 ± 0.11^a^	6.82 ± 2.74^b^	0.59 ± 0.83^c^	0.61 ± 1.13^b^	1.69 ± 3.08^c^	5.47 ± 0.52^b^	3.27 ± 1.23^a^	2.14 ± 0.31^b^	0.17 ± 0.11^c^	0.07 ± 0.003^a^
HFDC		2.67 ± 0.97^c^	4.66 ± 1.60^c^	14.30 ± 8.2^d^	1.82 ± 0.99^d^	1.55 ± 0.85^c^	5.07 ± 2.48^d^	18.35 ± 0.45 ^c^	18.52 ± 0.49^d^	5.77 ± 2.76^c^	0.42 ± 0.15^d^	0.25 ± 0.09^b^
HFD + MCC2759		0.83 ± 0.71^b^	1.62 ± 1.53^b^	7.70 ± 0.06^c^	0.41 ± 0.26^b^	0.56 ± 0.63^b^	1.57 ± 0.84^b^	4.36 ± 0.71^a^	6.53 ± 4.27^c^	1.54 ± 0.39^a^	0.15 ± 0.15^b^	0.06 ± 0.01^a^
HFD + MCC2760		0.57 ± 0.07^a^	1.17 ± 0.19^a^	5.13 ± 0.49^a^	0.35 ± 0.10^a^	0.42 ± 0.05^a^	1.27 ± 0.13^a^	4.33 ± 0.42^a^	4.29 ± 0.87^b^	1.58 ± 0.31^a^	0.09 ± 0.01^a^	0.05 ± 0.01^a^
Diabetic study 2												
NC		0.77 ± 0.09 ^c^	1.20 ± 0.09 ^c^	6.82 ± 0.44 ^c^	0.59 ± 0.28 ^c^	0.61 ± 0.11 ^c^	1.69 ± 0.44 ^a^	5.47 ± 1.07 ^c^	3.27 ± 1.00 ^b^	2.14 ± 0.62 ^c^	0.26 ± 0.12 ^c^	0.21 ± 0.14 ^c^
DC		0.55 ± 0.21 ^b^	0.90 ± 0.05 ^a^	4.76 ± 0.88 ^a^	0.30 ± 0.05 ^b^	0.19 ± 0.07 ^a^	2.41 ± 0.07 ^c^	5.33 ± 0.56 ^b^	4.81 ± 1.03 ^d^	0.93 ± 0.12 ^a^	0.093 ± 0.019 ^b^	0.05 ± 0.009 ^b^
D + MCC2759		0.46 ± 0.13 ^a^	1.03 ± 0.12 ^b^	5.11 ± 0.42 ^b^	0.39 ± 0.13 ^b^	0.26 ± 0.08 ^b^	2.14 ± 0.52 ^b^	4.57 ± 1.13 ^a^	4.39 ± 2.39 ^c^	0.97 ± 0.15 ^a^	0.089 ± 0.019 ^b^	0.058 ± 0.003 ^b^
D + MCC2760		0.48 ± 0.05^a^	0.97 ± 0.07 ^b^	4.57 ± 0.25 ^a^	0.24 ± 0.06 ^a^	0.22 ± 0.06 ^b^	2.43 ± 0.32 ^c^	5.78 ± 1.06 ^d^	3.07 ± 0.79 ^a^	1.44 ± 0.49 ^b^	0.074 ± 0.001 ^a^	0.04 ± 0.004 ^a^

Diabetes induction resulted in weight loss of the rats compared with the NC group. The bodyweight of *Lact. fermentum* treated groups did not significantly differ from the DC rats. Diabetes-induced rats also showed typical symptoms of increased urination, increased food, and water intake. There was not much significant difference in the food intake of *Lact. fermentum* treated groups compared with the diabetic control group (30.3 g/24 h at 4th week) although it was slightly reduced by the end of 4th week (25–27 g/24 h at 4th week) (Supplementary data 3). The weight of different organs such as the heart, liver, pancreas, and stomach significantly reduced in diabetic rats compared with normal rats. The weight of the kidney was significantly higher in diabetic rats than the non-diabetic rats (Table 1).

### Oral Glucose Tolerance and Plasma Insulin

**Fig. 1:**
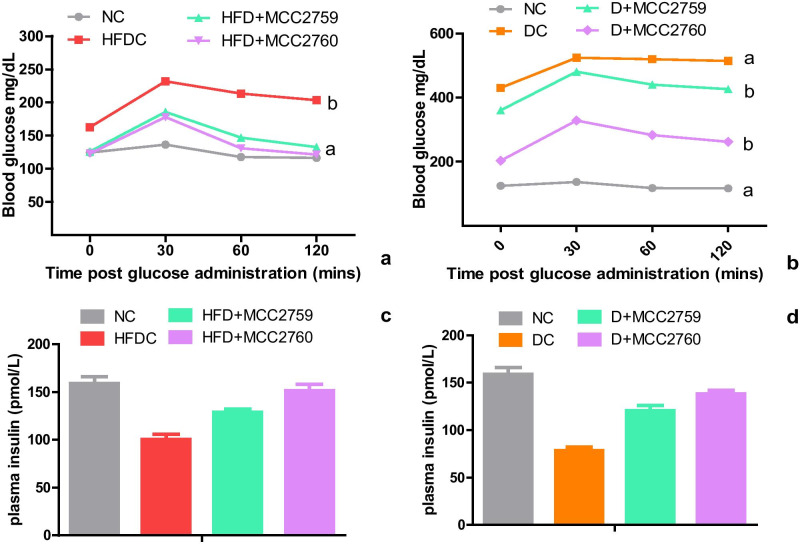
Effect of potential probiotic administration on **a** and **b** oral glucose tolerance test and **c** and **d** plasma insulin (pmol/l) in high-fat diet and STZ-induced diabetic rat groups Data presented as mean ± SEM (*n* = 6). NC normal control, DC diabetic control, D diabetic, HFDC high-fat diet control, HFD high-fat diet, MCC2759 and MCC2760 *L. fermentum* spp

OGTT of HFD (study 1) and STZ diabetic study (study 2) after 8 weeks and 4 weeks is depicted in Fig. 1. The data in Fig. 1 a and b is presented as blood glucose values (mg/dL) plotted against time post glucose administration. To determine the glucose tolerance, we calculated the AUC, which is widely used as an index for glucose excursion. DC and HFDC groups showed poor glucose tolerance with AUC 628.8 and AUC 1518, respectively. *Lact. fermentum* treatment groups showed lower AUC values suggesting improved glucose tolerance compared with model control groups in both studies. The AUC of the normal control group was 374.8. Plasma insulin levels in study 1 were 159 ± 9.89 and 100.5 ± 7.77 pmol/L for the NC and HFDC group, respectively (Fig. 1). The values improved in the *Lact. fermentum* treated groups of HFD + MCC2759 (129 ± 4.24 pmol/L) and HFD + MCC2760 (151.5 ± 9.19 pmol/L). In study 2, the lowest levels of plasma insulin were seen in the DC group (78.5 ± 4.94 pmol/L), while the *Lact. fermentum* treated groups seemed to normalize compared with the NC group with about 120.5 ± 7.77 and 138 ± 5.65 pmol/L for D + MCC2759 and D + MCC2760 groups, respectively (Fig. 1).

### Serum Biochemical Activity

Serum biochemical analysis was done to evaluate the effect of potential probiotic treatment on biochemical markers (Table 2). In the high-fat diet study 1, there was an increase in the levels of cholesterol (126.75 mg/dL), LDL-C (163.17 mg/dL), triglycerides (139.93 mg/dL), and glucose (160.13 mg/dL) in the high-fat diet control group as a result of fatty diet consumption. However, the levels were normalized significantly in the groups that received potential probiotic bacteria by the end of the experimental period.

**Table 2:** Serum biochemical changes of diabetic study groups and high-fat diet study groups

Parameters	Baseline levels	NC	Groups^1^					
			High-fat diet-fed study 1			Diabetic study 2		
			HFDC	HFD + MCC2759	HFD + MCC2760	DC	D + MCC2759	D + MCC2760
LDL (mg/dL)	94.67 ± 0.39	94.87 ± 0.05^a^	163.17 ± 10.50^d^	154.43 ± 0.62^c^	136.19 ± 10.50^b^	157.63 ± 7.09^c^	117.37 ± 1.16^b^	117.04 ± 1.46^b^
HDL (mg/dL)	54.01 ± 0.18	53.46 ± 1.34^a^	48.63 ± 3.45^a^	50.42 ± 3.69^b^	52.52 ± 7.44^b^	50.77 ± 6.09^a^	61.51 ± 3.18^b^	68.04 ± 8.37^c^
Cholesterol (mg/dL)	62.22 ± 0.22	63.57 ± 9.23^a^	126.75 ± 1.69 ^d^	84.31 ± 2.19^c^	60.62 ± 3.23^a^	90.80 ± 8.99^b^	89.34 ± 2.75^b^	64.53 ± 8.99^a^
Triglycerides (mg/dL)	79.95 ± 0.20	79.38 ± 2.73^b^	139.93 ± 10.91^d^	81.44 ± 0.77^c^	54.65 ± 8.11^a^	143.32 ± 2.7^d^	90.18 ± 3.00^c^	68.46 ± 7.63^a^
Total Protein (g/dL)	7.13 ± 0.11	6.26 ± 1.06^c^	7.81 ± 0.18^c^	8.95 ± 0.79^d^	4.78 ± 0.22^a^	5.95 ± 0.13^a^	6.17 ± 0.90^b^	6.84 ± 1.29^d^
Uric acid (mg/dL)	4.18 ± 0.36	3.64 ± 0.57^a^	2.56 ± 0.19^b^	2.48 ± 1.00^a^	3.18 ± 0.94^c^	7.34 ± 3.80^b^	8.88 ± 6.41^d^	8.39 ± 1.55^c^
Urea (mg/dL)	25.38 ± 0.11	24.89 ± 8.40^a^	10.96 ± 1.01^b^	10.38 ± 2.34^a^	11.18 ± 0.42^c^	52.05 ± 1.16^c^	40.34 ± 7.47^b^	36.20 ± 7.70^b^
Creatinine (mg/dL)	0.84 ± 0.05	0.61 ± 1.85^a^	0.55 ± 0.008^b^	0.50 ± 0.04^a^	0.56 ± 0.02^b^	1.11 ± 0.05^d^	0.75 ± 0.01^b^	1.18 ± 0.10^c^
Glucose (mg/dL)	119.60 ± 0.31	121.27 ± 8.30^a^	160.13 ± 13.51^c^	109.05 ± 6.73^a^	105.49 ± 5.49^a^	436.10 ± 5.88^d^	367.71 ± 2.42^c^	201.72 ± 5.21^b^
Albumin (g/dL)	4.39 ± 0.18	3.89 ± 0.71^c^	2.40 ± 0.33^a^	3.66 ± 0.37^c^	3.07 ± 0.58^b^	3.44 ± 0.45^a^	3.52 ± 0.12^b^	3.51 ± 0.21^b^
SGPT (U/L)	38.83 ± 0.17	39.59 ± 0.02^c^	26.95 ± 0.36^b^	20.88 ± 2.40^a^	21.23 ± 3.08^a^	18.31 ± 4.19^a^	28.79 ± 7.15^b^	41.17 ± 1.07^d^
SGOT (U/L)	46.05 ± 0.23	45.56 ± 0.03^b^	42.39 ± 3.06^b^	29.68 ± 0.61^a^	42.75 ± 3.57^b^	45.02 ± 0.04^b^	49.89 ± 0.08^c^	39.26 ± 0.12^a^

^1^Values represented as mean ± SD (*n* = 6). Values with different superscripts are significant (*p* < 0.05)

^2^*LDL* low-density lipoprotein cholesterol, *HDL* high-density lipoprotein cholesterol, *SGPT* serum glutamate-pyruvate transaminase, *SGOT* serum glutamic oxaloacetic transaminase, *D* diabetic, *MCC2759 and MCC2760* *L. fermentum* spp

In study 2, levels of cholesterol, triglycerides, and LDL-C were significantly lower in the *Lact. fermentum* treated groups compared with the diabetic control group. Serum glucose levels significantly reduced upon treatment with *Lact. fermentum* MCC2759 (367.71 mg/dL) and MCC2760 (201.72 mg/dL) respectively, compared with elevated levels in diabetic control group (436.10 mg/dL).

### Effect of *Lact. fermentum* Administration on Gene Expression

**Fig. 2:**
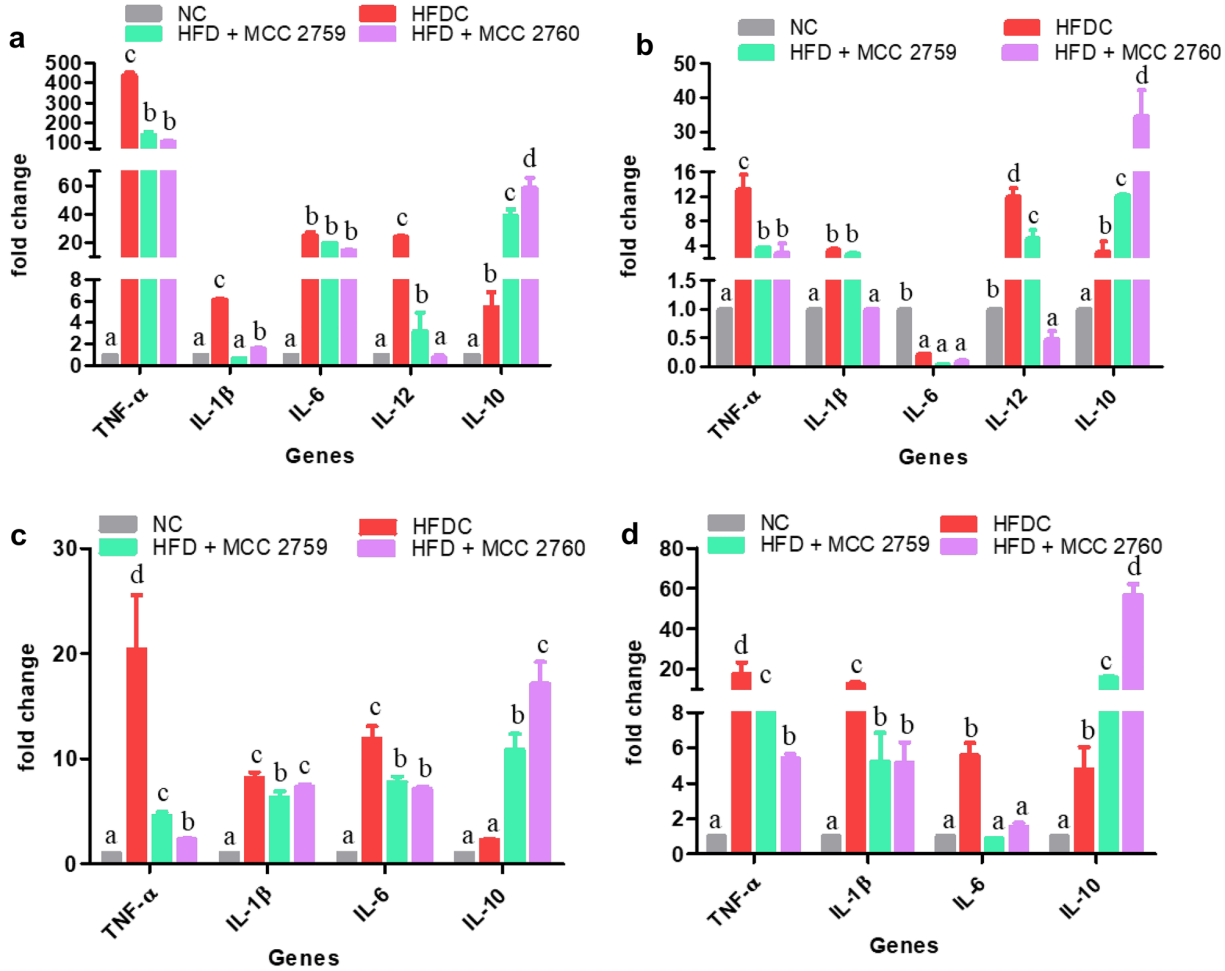
Expression changes in inflammatory markers of different high-fat diet fed study groups monitored by qPCR **a** intestine, **b** liver, **c** MAT and **d** muscle tissue; Data presented as mean ± SEM (*n* = 6). Letters with different superscripts are significant at *p* < 0.001. The mRNA expression was normalized to GAPDH. NC normal control, HFDC high-fat diet control, HFD high-fat diet, MCC2759 and MCC2760 *L. fermentum* spp

**Fig. 3:**
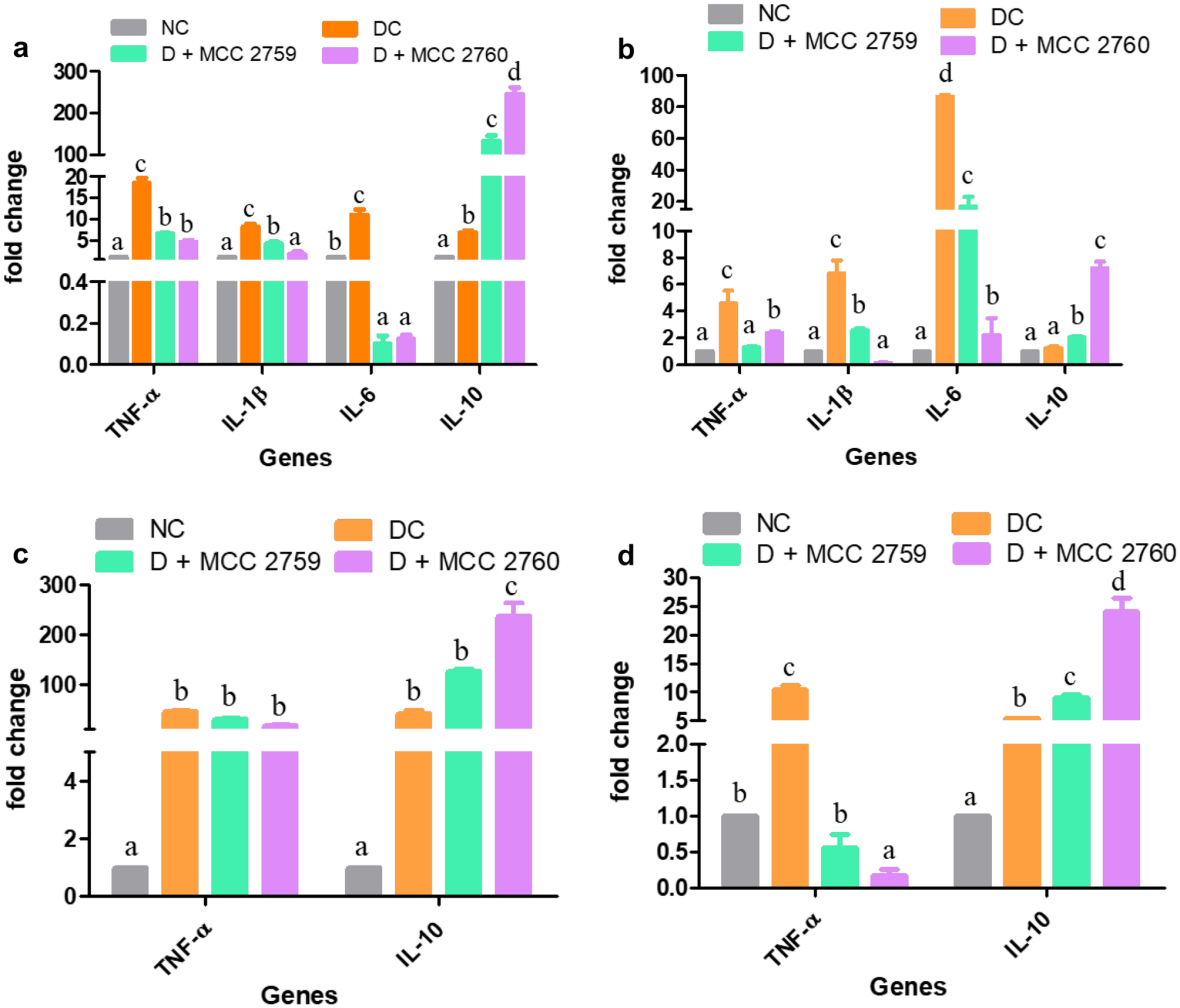
Expression changes in inflammatory markers of different diabetic study groups monitored by qPCR: **a** intestine, **b** liver, **c** MAT and **d** muscle tissue. Data presented as mean ± SEM (*n* = 6). Letters with different superscripts are significant at *p* < 0.001. The mRNA expression was normalized to GAPDH. NC normal control, DC diabetic control, D diabetic, MCC2759 and MCC2760 *L. fermentum* spp

Relative gene expression of inflammatory markers such as TNF-α, IL-1β, IL-6, and IL-10 in tissues such as intestine, liver, MAT, and muscle are presented in Fig. 2 for study 1 and Fig. 3 for study 2. The expression levels of pro-inflammatory markers TNF-α, IL-1β, and IL-6 were elevated in the HFD control group (study 1) and diabetic control group (study 2) in the tissues. However, groups treated with *Lact. fermentum* showed downregulation of TNF-α while inducing the expression of anti-inflammatory marker IL-10 in both studies. While IL-6 and IL-1β expression was detected in adipose and muscle tissues in study 1, we were unable to detect the same in study 2.

**Fig. 4:**
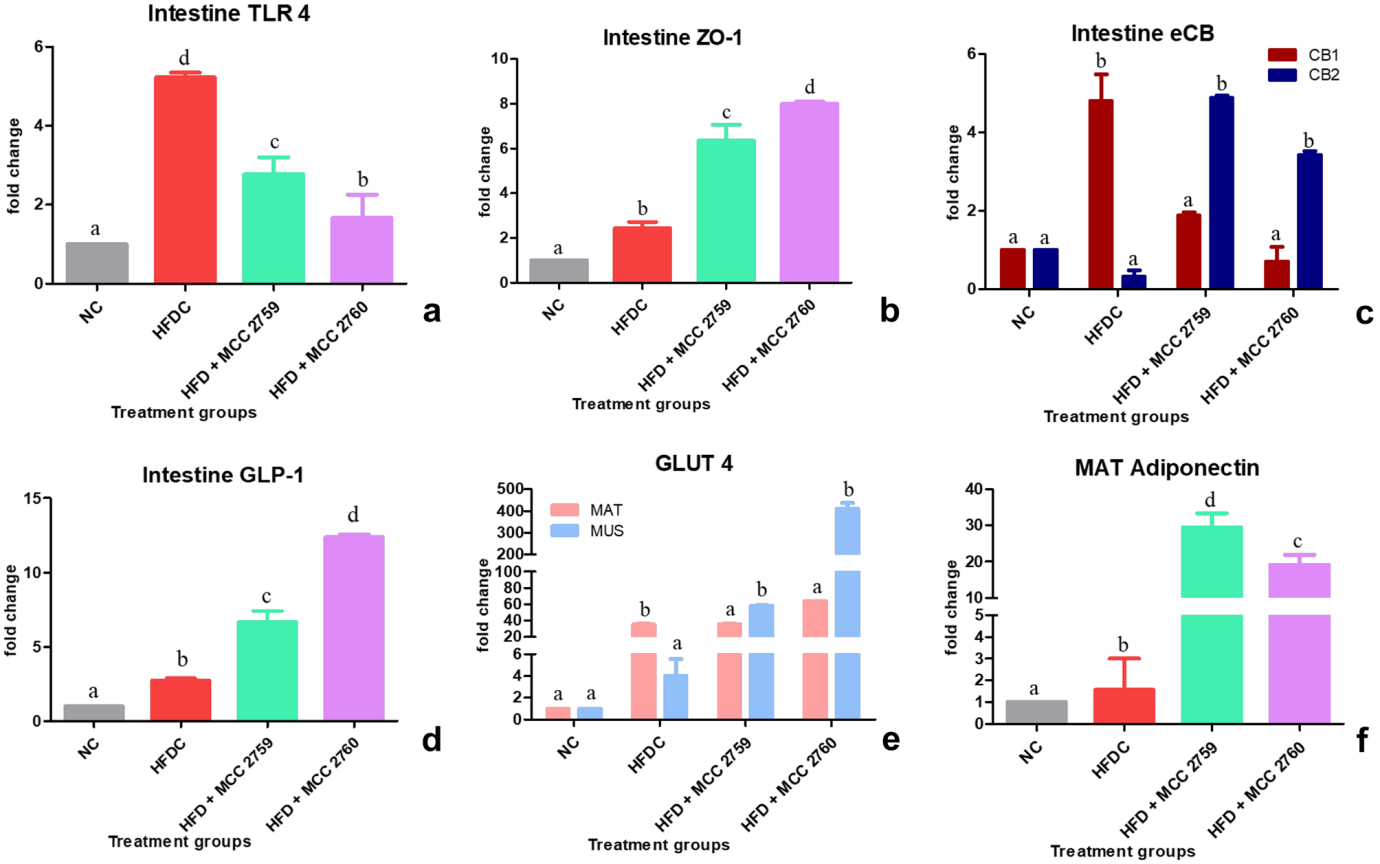
Expression changes in intestinal and other markers in high-fat diet fed study groups monitored by qPCR: **a** TLR4, **b** ZO-1, **c** eCB system, **d** GLP1 intestinal markers, **e** GLUT4 expression levels in mesenteric adipose tissue and muscle tissue and **f** adiponectin expression levels in mesenteric adipose tissue. Data presented as mean ± SEM (*n* = 6). Letters with different superscripts are significant at *p* < 0.001. The mRNA expression was normalized to GAPDH. NC normal control, HFDC-high-fat diet control, HFD high-fat diet, MCC2759 and MCC2760 *L. fermentum* spp., MAT mesenteric adipose tissue, MUS muscle

**Fig. 5:**
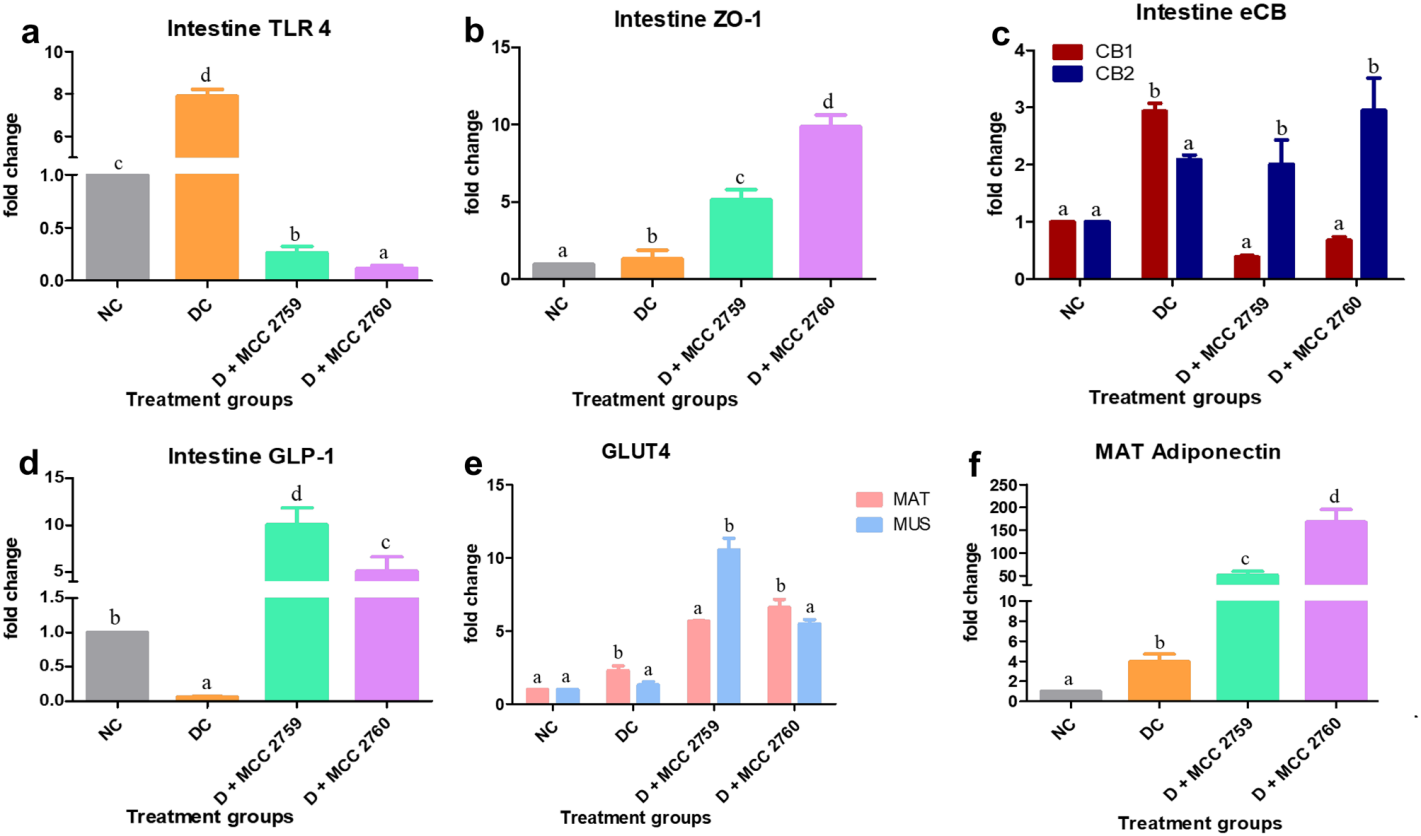
Expression changes in intestinal and other markers in diabetic study groups monitored by qPCR: **a** TLR4, **b** ZO-1, **c** eCB system, **d** GLP1 intestinal markers, **e** GLUT4 expression levels in mesenteric adipose tissue and muscle tissue and **f** adiponectin expression levels in mesenteric adipose tissue. Data presented as mean ± SEM (*n* = 6). Letters with different superscripts are significant at *p* < 0.001. The mRNA expression was normalized to GAPDH. NC normal control, DC diabetic control, D diabetic, MCC2759 and MCC2760 *L. fermentum* spp., MAT mesenteric adipose tissue, MUS muscle

*Lact. fermentum* MCC2759 and MCC2760 treated groups exhibited significant reduced expression of the TLR4 receptor compared with control groups of both studies. Expression of tight junction protein ZO-1, endocannabinoid receptor CB2 and glucagon-like peptide 1 (GLP1) were also increased in the *Lact. fermentum* treated groups (Figs. 4 and 5). In MAT and muscle tissue, *Lact. fermentum* treated groups exhibited improved expression of GLUT4 in both studies. Additionally, the expression of adiponectin was upregulated in treatment groups compared with HFD, and STZ-induced diabetic control groups.

### Histopathological Observations

In the high-fat diet-fed study, liver sections stained with hematoxylin and eosin showed cellular infiltration of neutrophils suggesting initial inflammatory response and formation of few vesicular structures indicating the initiation of hepatic steatosis. However, both the inflammatory infiltration and steatosis formation were reversed in the groups treated with *Lact. fermentum* MCC2759 and MCC2760. Slight disruptions were observed in intestinal sections of high-fat diet-fed rats showing mucosal damage and irregular crypts which were normalized in the *Lact. fermentum* treated groups while slight glomerular darkening was observed in kidney tissues.

**Fig. 6:**
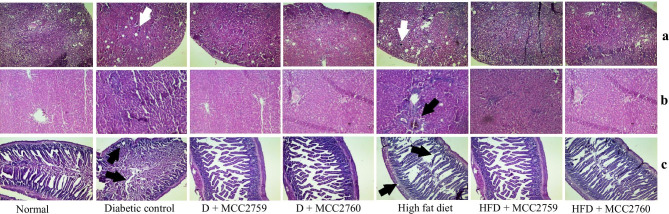
Histopathological indications of control and *Lact. fermentum* treated groups in diabetic and high-fat diet fed study. **a** Liver tissue—arrow indicates neutrophil infiltration; **b** kidney tissue—arrows indicate darkened glomeruli; **c** intestine—arrows indicate irregular crypts, damage of epithelial surface and shedding of the smooth muscle lining; hematoxylin and eosin staining at × 40 magnification; NC normal control, DC diabetic control, D diabetic, HFDC high-fat diet control, HFD high-fat diet, MCC2759 and MCC2760 *L. fermentum* spp

Liver micrographs of diabetic study rats showed reduced inflammation in the *Lact. fermentum* treated groups compared with the diabetic control group which showed accumulation of neutrophils around the portal tracts. Kidney sections showed a reduction in the glomerular injury of diabetic rats characterized by darkened Bowman’s capsule and thickening of the capsule after treatment with *Lact. fermentum* spp. Diabetic rats also showed disruptions in the intestinal structure showing damage of mucosa, crypts, and thinning of the muscle lining with shedding. However, *Lact. fermentum* treatment normalized the integrity of the intestinal mucosa with more goblet cells. Histopathological changes in both models are presented in Fig. 6.

## Discussion

There is an increasing demand for indigenous probiotic cultures with strain-specific health benefits against various lifestyle diseases. Thus, the isolates *Lact. fermentum* MCC2759 (infant faecal isolate) and *Lact. fermentum* MCC2760 (dairy isolate) were selected based on their potential probiotic properties and anti-inflammatory activity [13–15]. In this study, we evaluated the efficacy of these two isolates in alleviating the effects of HFD-induced pre-diabetic inflammatory condition as well as the effects of type 2 diabetes. High-fat diet fed for three or more weeks is said to induce insulin resistance and initiate a low-grade inflammatory tone in important organs of the body making the host highly susceptible to attaining type 2 diabetes. Conversely, HFD coupled with a low dose of STZ is said to mimic the metabolic characteristics of type 2 diabetes and is a novel model for anti-diabetic studies [12].

High-fat or high-cholesterol diet contributes to an increase in body weight. Few studies have reported the weight-lowering ability of lactic acid bacteria (LAB) in such models. Certain strains of LAB have shown the ability to prevent body weight gain [16, 17]. Increased excretion of bile acids in the faeces may increase bile acid synthesis and induce energy expenditure contributing to weight loss. In study 1, due to HFD feeding, the control groups gained a significant amount of weight throughout the 8 weeks. However, the weight gain and food intake were significantly controlled in groups that received *Lact. fermentum* MCC2759 and MCC2760 from the 5th week. STZ-induced diabetes typically presents severe loss of body weight due to degeneration of structural proteins that are crucial for body weight [12, 18]. There was no significant increase in the bodyweight of *Lact. fermentum* treated groups compared with the diabetic control group in study 1 implying the effects of STZ. Reduced body weight also corresponded to the reduced organ weight in study 2 groups. In correlation with body weight, serum biochemical markers such as glucose, triglycerides, and cholesterol were improved in *Lact. fermentum* treated groups in both studies. Similar positive effects of probiotics on serum markers were also found in other studies [19–21]. Some strains of probiotics are known to reduce serum cholesterol levels by increasing the excretion of bile acids in faeces and affecting cholesterol synthesis pathways [22, 23].

Obesity and diabetes are metabolic disorders characterized by a low-grade inflammatory condition [6]. TNF-α is a pro-inflammatory cytokine which under elevated conditions is known to phosphorylate serine residue substrate (IRS-1) on the insulin receptor causing its inactivation, while IL-1β, TNF-α, and IFN-γ are known to act synergistically by infiltrating the pancreas and inducing β-cell damage and apoptosis [24]. Overall the raised levels of these pro-inflammatory cytokines in hepatocytes, muscle, and adipose tissue play a major role in the pathology of diabetes by interfering with insulin signalling and inducing insulin resistance. However, supplementation of *Lact. fermentum* showed downregulation of these genes in the intestine, liver, MAT, and muscle mainly by stimulating the expression of anti-inflammatory regulatory cytokine IL-10 suggesting its immunomodulatory potential. *Lactobacillus casei *strain *Shirota* showed a significant reduction in the levels of pro-inflammatory cytokines IL-6, IL-4, and C-reactive protein in STZ-induced diabetic rats [25]. Probiotic mixture VSL#3 was found to reduce the levels of TNF-α and concomitantly augment IL-10 in a diabetic mice model fed for 12 weeks [26]. Similar studies have shown the effects of probiotic lactobacilli on inflammatory markers [20, 27]. Our findings are consistent with previous reports suggesting the anti-inflammatory potential of *Lact. fermentum* spp.

Microbial detection in the intestine occurs by recognition of pathogen-associated molecular patterns (PAMPs) such as LPS by specialized receptors called TLRs present on the cell surface that trigger the immune response [28]. Constant levels of LPS in the gut trigger an inflammatory response via the TLR4. In both models, the expression of TLR4 was found to be down-regulated by *Lact. fermentum* treatment suggestive of its ability to modulate gut microbiota and control the levels of LPS generated. The eCB system is also thought to modulate intestinal permeability by enhancing the distribution and localization of tight junction proteins [29]. In our study, *Lact. fermentum* displayed the ability to influence the eCB system by down-regulating the expression of CB1 and augmenting the expression of CB2, thereby reducing the gut LPS concentrations and improving barrier function. The enhanced barrier functionality can also be evident in the enhanced ZO-1 expression by *L. fermentum* treatment. Species of *Lactobacillus*, namely *Lact. acidophilus*, *Lact. rhamnosus*, *Lact. gasseri*, and* Lact. fermentum*, is known to influence the expression of tight junction proteins [30]. Supplementation of *Lact. fermentum* augmented the expression of GLP-1 in the intestine which seems to play a role in improving inflammation and insulin resistance caused by high-fat diet intake [6]. Probiotics are known to influence the secretion of GLUT4, thereby facilitating glucose uptake and reduction of insulin resistance in adipose and muscle tissue [31]. Kim et al. [32] showed that *Lact. rhamnosus* GG stimulated GLUT4 expression in muscle tissue of high-fat diet-fed mice. In our study, *Lact. fermentum* supplementation also positively influenced the expression of GLUT4 in MAT and muscle tissue correlating with previous reports. *Lact. fermentum* administration to the diabetic and high-fat diet-fed rats resulted in higher expression of adiponectin levels probably due to the reduction of pro-inflammatory response in adipose tissue and enhancement of GLUT4 [32]. Adiponectin levels are said to be low in obese subjects [33]. Probiotic therapy may help in augmenting their levels by stimulating GLUT4 and reducing inflammation in adipose tissue.

Insulin resistance in the cells of different tissues is marked by the cellular infiltration of inflammatory molecules due to enhanced secretion of pro-inflammatory cytokines. Infiltration of neutrophils indicates inflammation of the tissues as evident by qPCR results which showed increased expression of pro-inflammatory cytokines. *Lact. fermentum* administration in both studies exhibited reduced neutrophil infiltration as seen in histopathological observations. Oral administration of *Lact. reuteri* improved hepatic steatosis and insulin resistance in liver tissues of high fructose-fed rats [34]. These findings indicate that inflammation and steatosis caused due to the consumption of a high-fat diet may be ameliorated by the administration of *Lact. fermentum.* Consumption of a high-fat diet causes dysbiosis of the intestinal mucosal milieu compromising the integrity of the mucosal barrier. *Lact. fermentum* treated groups showed improved mucosal lining and barrier integrity structure compared with the control. Darkened and diffused sections of Bowman’s capsule in the kidney indicate macrophage infiltration as previously observed by Sharavana et al. [35], which normalized upon *Lact. fermentum* treatment in our study.

In conclusion, this study demonstrated the beneficial role of *Lact. fermentum* spp. principally through its anti-inflammatory and potential probiotic action in models of HFD feeding and STZ-induced diabetes. *Lact. fermentum* MCC2759 and MCC2760 exhibited beneficial effects in HFD and type 2 diabetic models by improving glucose and lipid profile, reduction of pro-inflammatory cytokines in liver, intestine, MAT, and muscle tissue, and improving the intestinal barrier function and expression of GLUT4, GLP1, and adiponectin. *Lact. fermentum* MCC2760 showed slightly better effects compared with MCC2759. The cultures could act as promising candidates for inflammatory conditions as they show an overall reduction of inflammation in various tissues and organs of the body. Count of pathogenic bacteria like *Escherichia coli*, *Staphylococcus aureus*, and *Campylobacter* spp. appeared to be reduced in faeces of *Lact. fermentum* treated groups of both studies as obtained by plate count (data not shown). However, further studies and human trials are required to validate their effects.

## Data Availability 

The datasets generated during and/or analysed during the current study are available from the corresponding author on reasonable request.

## Supplementary Information

Below is the link to the electronic supplementary material.

Supplementary file1 (PDF 203 KB)

Supplementary file2 (PDF 410 KB)

Supplementary file3 (PDF 214 KB)
