# Supplementary material for: Lactobacillus fermentum MCC2759 and MCC2760 Alleviate Inflammation and Intestinal Function in High-Fat Diet-Fed and Streptozotocin-Induced Diabetic Rats
Source: Probiotics Antimicrob Proteins. 2021 Feb 11;13(4):1068–80. doi: 10.1007/s12602-021-09744-0 (PMC8342349; doi:10.1007/s12602-021-09744-0)
Supplement: Supplementary file 1 — Supplementary file1 (PDF 203 KB) [file 12602_2021_9744_MOESM1_ESM.pdf]

Lactobacillus fermentum MCC2759 and MCC2760 alleviate inflammation and intestinal function in high-fat diet-fed and streptozotocin-induced diabetic rats

Ann Catherine Archer[1], Serva Peddha Muthukumar[2], Prakash Motiram Halami[1*]

1Microbiology and Fermentation Technology Department,
2Department of Biochemistry,
CSIR-Central Food Technological Research Institute, Mysuru-570020, India

*Corresponding author
Mailing address: Microbiology and Fermentation Technology Department,
CSIR-Central Food Technological Research Institute, Mysuru-570020, India.
Phone: +91-821-2517539 Fax: +91-821-2517233.
E-mail address: prakashalami@cftri.res.in (Halami PM).

Supplementary data 1: Composition of high fat diet (D12492, Research diets Inc.)

Ingredient	Weight in g/1000 g	Kcal
Casein	200	800
L-cysteine	3	12
Maltodextrin 10	125	500
Sucrose	68.8	275.2
Cellulose	50	0
Soybean oil	25	225
Lard	245	2205
Mineral mix	10	0
Dicalcium phosphate	13	0
Calcium carbonate	5.5	0
Potassium citrate	16.5	0
Vitamin mix	10	40
