# Supplementary material for: Lactobacillus fermentum MCC2759 and MCC2760 Alleviate Inflammation and Intestinal Function in High-Fat Diet-Fed and Streptozotocin-Induced Diabetic Rats
Source: Probiotics Antimicrob Proteins. 2021 Feb 11;13(4):1068–80. doi: 10.1007/s12602-021-09744-0 (PMC8342349; doi:10.1007/s12602-021-09744-0)
Supplement: Supplementary file 2 — Supplementary file2 (PDF 410 KB) [file 12602_2021_9744_MOESM2_ESM.pdf]

***Lactobacillus fermentum* MCC2759 and MCC2760 alleviate inflammation and intestinal function in high-fat diet-fed and streptozotocin-induced diabetic rats**

Ann Catherine Archer[1], Serva Peddha Muthukumar[2], Prakash Motiram Halami[1*]

1Microbiology and Fermentation Technology Department,
2Department of Biochemistry,
CSIR-Central Food Technological Research Institute, Mysuru-570020, India

*Corresponding author
Mailing address: Microbiology and Fermentation Technology Department,
CSIR-Central Food Technological Research Institute, Mysuru-570020, India.
Phone: +91-821-2517539 Fax: +91-821-2517233.
E-mail address: prakashalami@cftri.res.in (Halami PM).

Supplementary data 2: List of primers used in this study

Target gene	Primer pair	Primer Sequences (5’ → 3’)	Amplicon size (bp)	Annealing temperature (°C)	Purpose	Reference
TNF-α	RNTNF-αF<br>RNTNF-αR	CAGATGGGCTGTACCTTATC<br>AGAGGAGGCTGACTTTCT	125	60	anti-inflammatory	This study
IL-1β	RNIL-1βF<br>RNIL-1βR	CCTGTCCTGTGTGATGAAAG<br>AACTGTGCAGACTCAAACTC	147	60	anti-inflammatory	This study
IL-6	RNIL-6F<br>RNIL-6R	CCTTCTTGGGACTGATGTTG<br>AATTAAGCCTCCGACTTGTG	128	60	anti-inflammatory	This study
IL-12	RNIL-12F<br>RNIL-12R	CATCCAGCGCAAGAAAGA<br>GAATTGTAGTAGCGGTCCTG	144	60	anti-inflammatory	This study
IL-10	RNIL-10F<br>RNIL-10R	GGAGTGAAGACCAGCAAAGG<br>GGCAACCCAAGTAACCCTTA	155	60	anti-inflammatory	Distrutti et al. [54]
GAPDH	RNGAPDHF<br>RNGAPDHR	GCCTCCAAGGAGTAAGAAAC<br>GTCTGGGATGGAATTGTGAG	140	60	endogenous control	This study
TLR4	TLR4F<br>TLR4R	ATCATCCAGGAAGGCTTCCA<br>GCTGCCTCAGCAAGGACTTCT	180	60	probiotic MOA	Jilling et al. [55]
ZO-1	ZO-1F<br>ZO-1R	AGCGAAGCCACCTGAAGATA<br>GATGGCCAGCAGGAATATGT	139	60	probiotic MOA	This study
CB1	CB1F<br>CB1R	TTTCAAGCAAGGAGCACCCA<br>GGTACGGAAGGTGGTGTCTG	134	60	probiotic MOA	This study
CB2	CB2F<br>CB2R	TGATAGCCCGATAAGCCTTG<br>TGCTATGGGAGACACAGCAG	135	60	probiotic MOA	This study
ADIPO	ADIPOF<br>ADIPOR	CATTCCTGTCTGTACGAGTG<br>GGCAGGATTAAGAGGAACAG	99	60	probiotic MOA	This study
GLUT4	GLUT4F<br>GLUT4R	GTCCCTACGTCTTCCTTCTA<br>GTTTCACCTCCTGCTCTAAG	149	60	probiotic MOA	This study
GLP1	GLP1F<br>GLP1R	CAAGCTGTACCTGAGCATAG<br>GAATGGGCAAGCGTATGA	142	60	probiotic MOA	This study

TNF-tumour necrosis factor; IL-interleukin; GAPDH-glyceraldehyde phosphate dehydrogenase; TLR-toll-like receptor; MOA-mode of action; ZO-zonal occluden; CB-endocannabinoid receptor; ADIPO-adiponectin; GLUT-glucose transporter; GLP-glucagon-like peptide; MOA-mode of action
