# Supplementary material for: Lactobacillus fermentum MCC2759 and MCC2760 Alleviate Inflammation and Intestinal Function in High-Fat Diet-Fed and Streptozotocin-Induced Diabetic Rats
Source: Probiotics Antimicrob Proteins. 2021 Feb 11;13(4):1068–80. doi: 10.1007/s12602-021-09744-0 (PMC8342349; doi:10.1007/s12602-021-09744-0)

Lactobacillus fermentum MCC2759 and MCC2760 alleviate inflammation and intestinal function in high-fat diet-fed and streptozotocin-induced diabetic rats

Ann Catherine Archer[1], Serva Peddha Muthukumar[2], Prakash Motiram Halami[1*]

1Microbiology and Fermentation Technology Department,
2Department of Biochemistry,
CSIR-Central Food Technological Research Institute, Mysuru-570020, India

*Corresponding author
Mailing address: Microbiology and Fermentation Technology Department,
CSIR-Central Food Technological Research Institute, Mysuru-570020, India.
Phone: +91-821-2517539 Fax: +91-821-2517233.
E-mail address: prakashalami@cftri.res.in (Halami PM).

Supplementary data 3

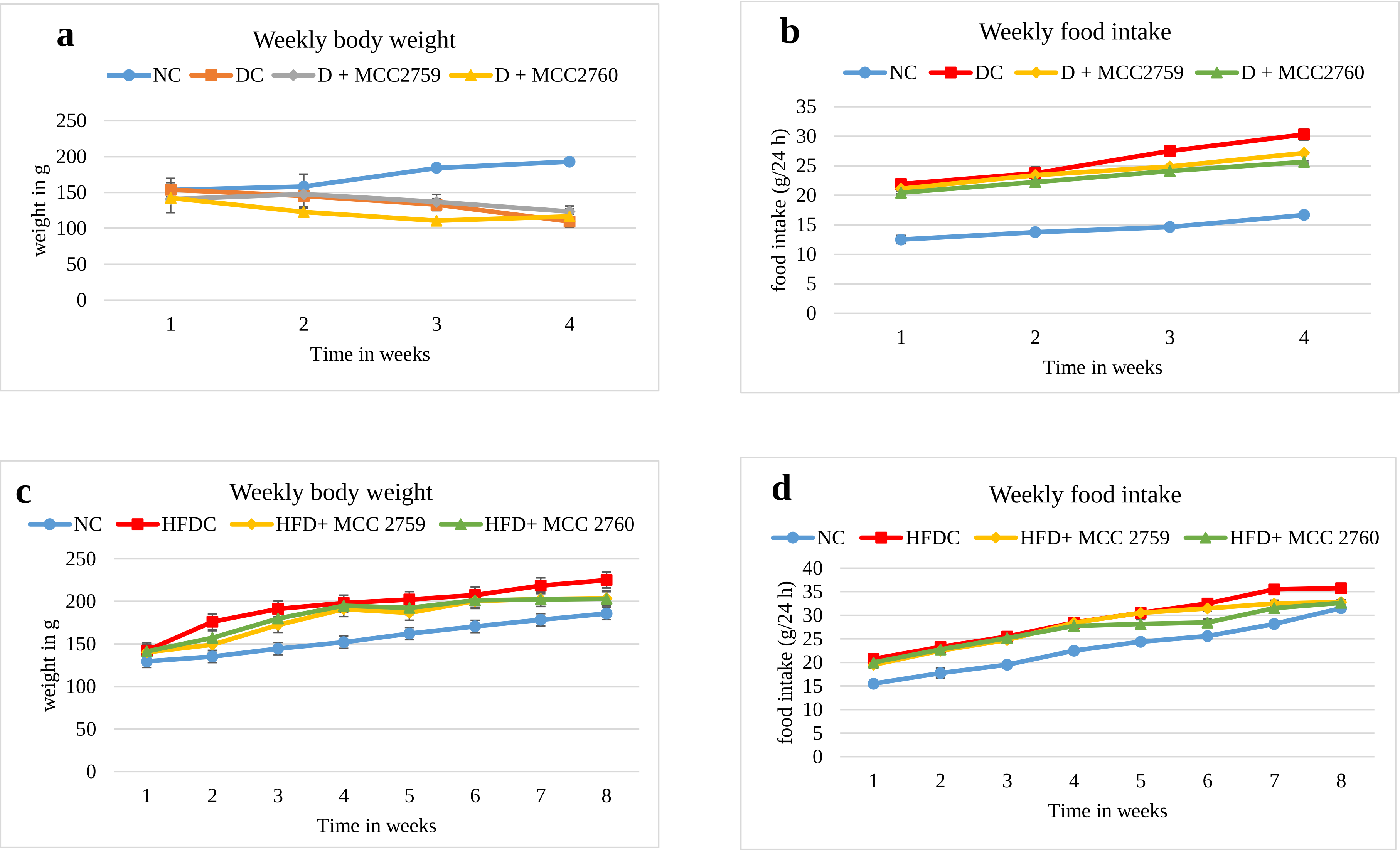

Supplement: Supplementary file 3 — Supplementary file3 (PDF 214 KB) [file 12602_2021_9744_MOESM3_ESM.pdf]
